# A Minimal Threshold of c-di-GMP Is Essential for Fruiting Body Formation and Sporulation in *Myxococcus xanthus*

**DOI:** 10.1371/journal.pgen.1006080

**Published:** 2016-05-23

**Authors:** Dorota Skotnicka, Gregory T. Smaldone, Tobias Petters, Eleftheria Trampari, Jennifer Liang, Volkhard Kaever, Jacob G. Malone, Mitchell Singer, Lotte Søgaard-Andersen

**Affiliations:** 1 Department of Ecophysiology, Max Planck Institute for Terrestrial Microbiology, Marburg, Germany; 2 Department of Microbiology and Molecular Genetics, University of California - Davis, Davis, California, United States of America; 3 Molecular Microbiology Department, John Innes Centre, Norwich, United Kingdom; 4 Research Core Unit Metabolomics, Hannover Medical School, Hannover, Germany; 5 School of Biological Sciences, University of East Anglia, Norwich, United Kingdom; Universidad de Sevilla, SPAIN

## Abstract

Generally, the second messenger bis-(3’-5’)-cyclic dimeric GMP (c-di-GMP) regulates the switch between motile and sessile lifestyles in bacteria. Here, we show that c-di-GMP is an essential regulator of multicellular development in the social bacterium *Myxococcus xanthus*. In response to starvation, *M*. *xanthus* initiates a developmental program that culminates in formation of spore-filled fruiting bodies. We show that c-di-GMP accumulates at elevated levels during development and that this increase is essential for completion of development whereas excess c-di-GMP does not interfere with development. MXAN3735 (renamed DmxB) is identified as a diguanylate cyclase that only functions during development and is responsible for this increased c-di-GMP accumulation. DmxB synthesis is induced in response to starvation, thereby restricting DmxB activity to development. DmxB is essential for development and functions downstream of the Dif chemosensory system to stimulate exopolysaccharide accumulation by inducing transcription of a subset of the genes encoding proteins involved in exopolysaccharide synthesis. The developmental defects in the *dmxB* mutant are non-cell autonomous and rescued by co-development with a strain proficient in exopolysaccharide synthesis, suggesting reduced exopolysaccharide accumulation as the causative defect in this mutant. The NtrC-like transcriptional regulator EpsI/Nla24, which is required for exopolysaccharide accumulation, is identified as a c-di-GMP receptor, and thus a putative target for DmxB generated c-di-GMP. Because DmxB can be—at least partially—functionally replaced by a heterologous diguanylate cyclase, these results altogether suggest a model in which a minimum threshold level of c-di-GMP is essential for the successful completion of multicellular development in *M*. *xanthus*.

## Author Summary

The nucleotide-based second messenger c-di-GMP is ubiquitous in bacteria and generally regulates the switch between motile and sessile lifestyles. We show that c-di-GMP regulates multicellular morphogenesis and cellular differentiation during the starvation-induced developmental program that culminates in fruiting body formation in *Myxococcus xanthus*. DmxB is identified as a diguanylate synthase that is regulated at multiple levels and responsible for the increased c-di-GMP synthesis during development. A Δ*dmxB* mutant has reduced exopolysaccharide accumulation and is rescued by co-development with strains proficient in exopolysaccharide accumulation, suggesting reduced exopolysaccharide accumulation as the causative defect in this mutant. We propose that a minimum threshold level of c-di-GMP is required for successful completion of starvation-induced development in *M*. *xanthus*.

## Introduction

Bacteria synthesize a variety of nucleotide-based second messengers that have important functions in adaptation and differentiation processes in response to environmental changes. Among these bis-(3’-5’)-cyclic dimeric GMP (c-di-GMP) has emerged as ubiquitous and highly versatile. Regulation by c-di-GMP often relates to lifestyle changes involving transitions between motility and sessility with the specific processes regulated including motility, adhesion, exopolysaccharide (EPS) synthesis, biofilm formation, cell cycle progression and virulence [for reviews, see [1–3]]. However, c-di-GMP also regulates multicellular development and cellular differentiation processes that do not appear to relate to this transition such as aerial mycelium formation in *Streptomyces venezuelae* and heterocyst formation in filaments of *Anabaena* sp. strain PCC 7120 [4, 5]. c-di-GMP is synthesized from two molecules of GTP by diguanylate cyclases (DGCs) [6, 7] that share the so-called GGDEF domain named after a conserved sequence motif in the active- (A) site [8]. GGDEF domains often contain an inhibitory-(I) site with the consensus sequence RxxD that binds c-di-GMP resulting in allosteric feedback inhibition of DGC activity [8]. c-di-GMP is degraded by phosphodiesterases (PDEs) that contain either a catalytic EAL or HD-GYP domain both of which are also named after conserved sequence motifs in their active site [9–12]. c-di-GMP interacts with a range of effectors to regulate downstream responses at the transcriptional, (post-)translational or allosteric level, as well as by mediating protein-protein interactions [1, 5, 13]. Effectors include degenerate GGDEF and EAL domain proteins that do not have catalytic activity [14–18], different transcription factor families [5, 19–24], PilZ domain-containing proteins [25–31], and riboswitches [32].

In response to nutrient starvation, the social bacterium *Myxococcus xanthus* initiates a multicellular developmental program that results in the formation of fruiting bodies that each contains ~100,000 spores [for review, see [33]]. Fruiting body formation proceeds in distinct morphological stages. After 4–6 hrs of starvation, the rod-shaped cells change motility behavior and start to aggregate to form translucent mounds [34]. By 24 hrs, the aggregation process is complete and those cells that have accumulated inside fruiting bodies differentiate to spherical spores with spore maturation completed by 72 hrs. While gliding motility is dispensable [35], type IV pili (T4P)-dependent motility is important for fruiting body formation and lack of T4P causes a delay or even blocks fruiting body formation while sporulation still proceeds [35, 36]. In *M*. *xanthus* T4P-dependent motility depends on EPS because it stimulates T4P retraction [37] and lack of EPS completely blocks fruiting body formation and sporulation [38–40]. EPS synthesis depends on the *eps* locus, which encodes structural proteins for EPS biosynthesis and transport [41]. Multiple regulators of EPS accumulation have been identified; however, only the NtrC-like transcriptional regulator EpsI/Nla24, which is encoded in the *eps* locus, and the Dif chemosensory system are essential for EPS synthesis [40–43]. Interestingly, lack of EPS causes a non-cell autonomous defect in development and development of mutants that lack EPS can be rescued by co-development with a strain proficient in EPS synthesis or by addition of purified EPS [38–40].

We recently demonstrated that *M*. *xanthus* accumulates c-di-GMP during growth and that c-di-GMP regulates T4P-dependent motility in growing cells by regulating transcription of the *pilA* gene, which encodes the major pilin of T4P, and EPS accumulation [44]. *M*. *xanthus* encodes 24 proteins containing a GGDEF, EAL or HD-GYP domain [15, 44]. A systematic genetic analysis using single gene mutations identified three of these genes (*dmxA*, *sgmT* and *tmoK*) as important for T4P-dependent motility whereas single mutations in the remaining 21 genes neither interfered with growth nor with motility [44]. Here, we identify c-di-GMP as an essential regulator of development in *M*. *xanthus* and show that reduced c-di-GMP levels cause a non-cell autonomous defect in EPS accumulation during development whereas increased c-di-GMP levels do not interfere with development. Moreover, we identify a novel DGC, DmxB, that only functions during development and is responsible for the increase in c-di-GMP levels that is essential for fruiting body formation and sporulation to go to completion. Moreover, we demonstrate that DmxB is essential for transcription of subset of genes involved in EPS synthesis and that the NtrC-like transcriptional regulator EpsI/Nla24 is a c-di-GMP receptor. Our results suggest a scenario in which a minimal threshold level of c-di-GMP is essential for progression of the developmental program in *M*. *xanthus* and binds to EpsI/Nla24 to stimulate EPS synthesis.

## Results

### c-di-GMP level increases significantly during development

**Fig 1:**
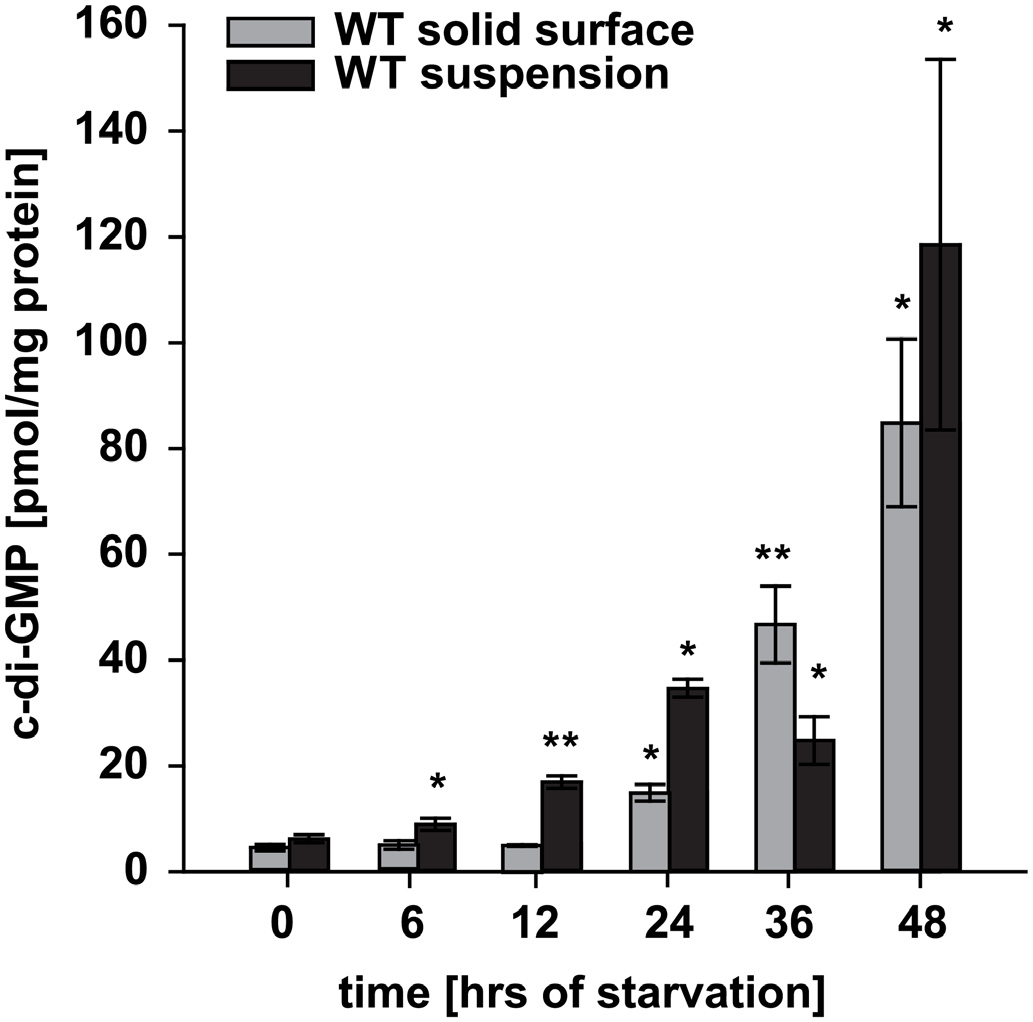
c-di-GMP accumulates at increased levels in developing and starving *M*. *xanthus* cells. DK1622 WT cells were starved in MC7 buffer on a solid surface in submerged culture or in suspension. At the indicated time points the c-di-GMP levels were determined and correlated to protein concentration. The c-di-GMP level is shown as mean ± standard deviation (SD) calculated from three biological replicates. * p < 0.05, ** p < 0.001 in Student’s t-test in which samples from individual time points were compared to the relevant 0 hrs sample.

We previously demonstrated that *M*. *xanthus* cells grown in suspension in rich medium accumulate c-di-GMP [44]. *M*. *xanthus* only forms fruiting bodies when starved on a surface. Therefore, to determine if *M*. *xanthus* accumulates c-di-GMP during development, exponentially growing wild-type (WT) DK1622 cells were removed from rich medium and starved on a solid surface in submerged culture for 48 hrs. c-di-GMP levels were quantified at different time points during development using liquid chromatography coupled tandem mass spectrometry [45]. c-di-GMP was detected at all time points of development. The c-di-GMP level increased ~20-fold from 0 hrs (4.4 ± 0.7 pmol/mg protein) to 48 hrs (84.8 ± 15.9 pmol/mg protein) of development (Fig 1). To determine if this increase in c-di-GMP was a specific response to starvation and not to a solid surface, cells were exposed to starvation in suspension. Under these conditions, the c-di-GMP level also increased ~20-fold from 0 hrs (6.2 ± 0.7 pmol/mg protein) to 48 hrs (118.5 ± 35.0 pmol/mg protein) of starvation (Fig 1). While the c-di-GMP level increased ~20-fold under both starvation conditions, the c-di-GMP level was generally lower in cells starved on a solid surface. Moreover, the c-di-GMP accumulation profile was slightly different, i.e. cells starved on a surface showed a significant increase in c-di-GMP after 24 hrs and cells starved in suspension a significant increase after 6 hrs of starvation. Importantly, because the level of c-di-GMP does not increase significantly in stationary phase cells [44], these data demonstrate that the increase in the c-di-GMP level is a specific response to starvation and that the c-di-GMP level increases during development. Because the c-di-GMP levels in suspension-starved WT cells overall correlated with that in developing WT, we from now measured c-di-GMP levels in cells starved in suspension.

### c-di-GMP level is important for fruiting body formation and sporulation

**Fig 2:**
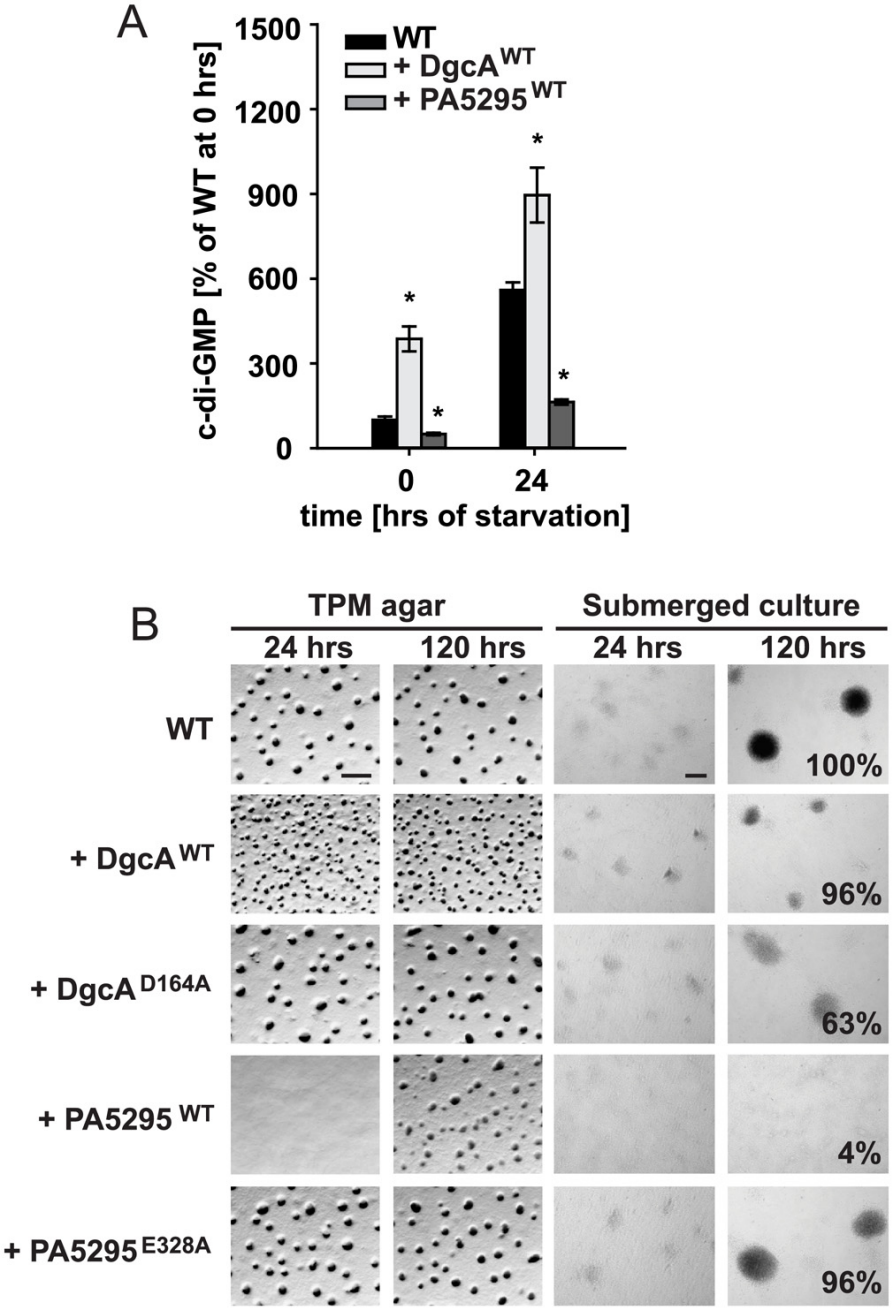
c-di-GMP level is important for development. (A) c-di-GMP levels in cells expressing the indicated proteins during starvation in suspension. The c-di-GMP levels are shown as mean ± SD from three biological replicates relative to WT at 0 hrs. * p < 0.05 in Student’s t-test comparing different mutants to the WT at the respective time points. Note that the data for WT are the same as in Fig 1. (B) Fruiting body formation and sporulation under two different starvation conditions. Numbers after 120 hrs of starvation in submerged culture indicate heat- and sonication resistant spores formed after 120 hrs of starvation in submerged culture in percentage of WT (100%) from one representative experiment. Scale bars, TPM agar 500 μm, submerged culture 100 μm.

To determine if the c-di-GMP level is important for development, we used previously generated strains [44] that constitutively produce a heterologous DGC (DgcA^WT^ of *Caulobacter crescentus*), a heterologous PDE (PA5295^WT^ of *Pseudomonas aeruginosa*) or their active site variants DgcA^D164A^ or PA5295^E328A^ in WT cells. Production of DgcA^WT^ and PA5295^WT^ causes a significant increase and decrease, respectively in the c-di-GMP level during vegetative growth whereas the two active site variants do not [44]. Consistently, after 24 hrs of starvation in suspension, the c-di-GMP level was significantly increased in cells producing DgcA^WT^ compared to WT and significantly decreased in cells expressing PA5295^WT^ (Fig 2A).

On TPM agar as well as in submerged culture, WT and the strains producing DgcA^D164A^ or PA5295^E328A^ aggregated to form nascent fruiting bodies after 24 hrs and had formed darkened spore-filled fruiting bodies after 120 hrs (Fig 2B). The strain producing DgcA^WT^ still formed fruiting bodies and sporulated but the fruiting bodies were smaller than in WT, possibly due to the defect that this strain has in T4P-dependent motility during vegetative growth [44]. By contrast, the PA5295^WT^ producing strain displayed delayed fruiting body formation on TPM agar, did not form fruiting bodies in submerged culture even after 120 hrs and its sporulation was strongly reduced. We conclude that a decreased c-di-GMP level impedes fruiting body formation and sporulation whereas an increased level of c-di-GMP does not.

### Identification of GGDEF, EAL and HD-GYP domain proteins important for development

We previously identified 24 genes in *M*. *xanthus* encoding proteins containing either a GGDEF (17 proteins), EAL (two proteins) or HD-GYP domain (five proteins) [15, 44]. Based on single gene mutations, three of these proteins are important for T4P-dependent motility: DmxA is a DGC, SgmT is a hybrid histidine protein kinase that binds c-di-GMP using its C-terminal degenerate GGDEF domain but does not have DGC activity, and TmoK is a hybrid histidine protein kinase with a C-terminal GGDEF domain that neither synthesizes nor binds c-di-GMP [15, 44].

To further investigate the function of these 24 proteins, we screened strains with single in-frame deletions in these genes or an insertion mutation in the case of *dmxA* for development-related phenotypes. Single gene mutations in 20 of the 24 genes did not affect development (S1 Fig). These 20 genes included *dmxA* and *actA*. ActA has previously been suggested to be important for development [46]. For generation of the in-frame deletion of *actA*, we reannotated *actA* taking into account the GC content in the third position of codons and by comparisons to orthologous genes (S2 Fig). Based on this re-annotation, the original Δ*actA* mutation extends into the promoter region of the *act* operon. *actA* is located upstream of *actB*, which is important for development (S2 Fig) [46]. Because the original Δ*actA* mutant phenocopies the Δ*actB* mutant, we speculate that the developmental defects observed for the original Δ*actA* mutant are caused by a polar effect on *actB*. We conclude that ActA is not required for development. Lack of the DGC DmxA did also not affect development and, thus, specifically causes a defect in T4P-dependent motility in vegetative cells.

As previously reported, lack of SgmT caused defects in fruiting body formation and sporulation [15]. Moreover, lack of TmoK caused delayed aggregation and reduced sporulation in submerged culture while aggregation was normal on TPM agar (S1 Fig).

**Fig 3:**
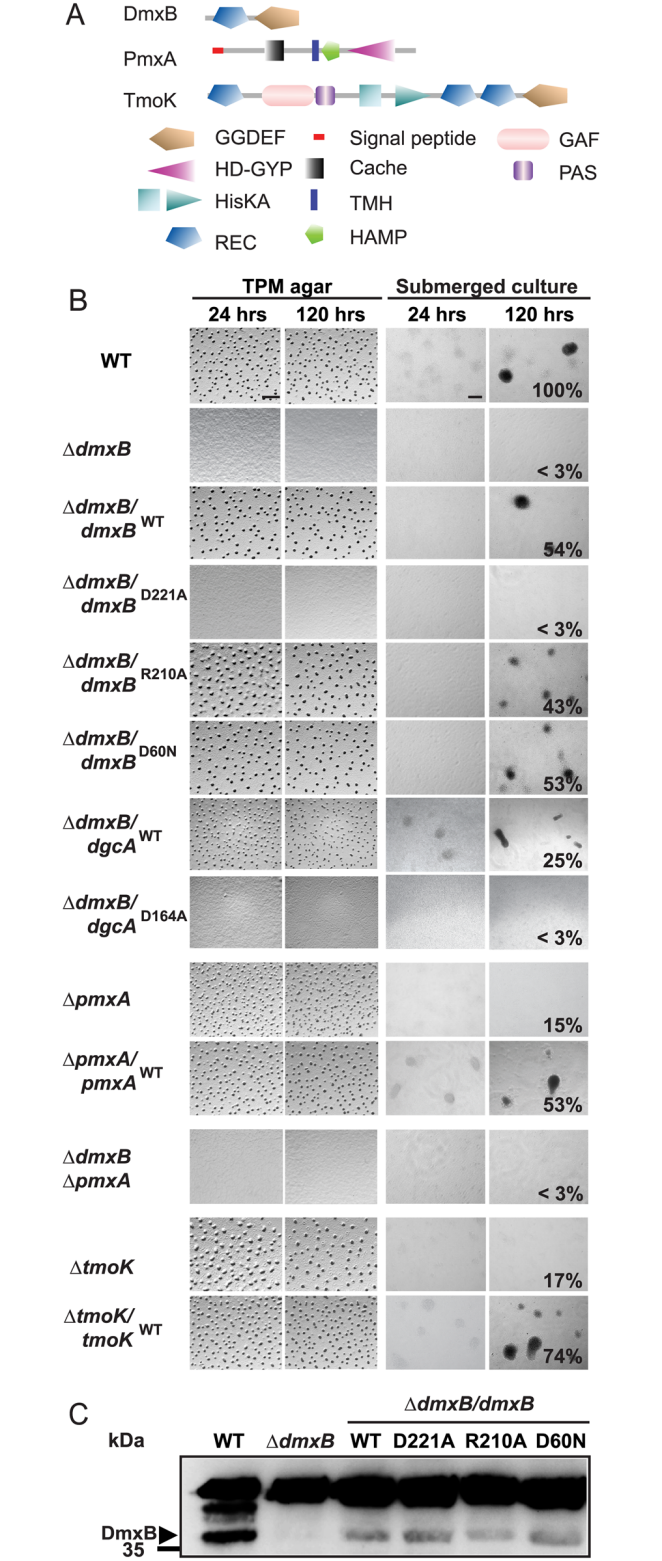
Complementation experiments with Δ*dmxB*, Δ*pmxA* and Δ*tmoK* mutants. (A) Domain structure of DmxB, PmxA and TmoK. The primary sequences of the indicated proteins were analyzed for domain structure using [47]. (B) Fruiting body formation and sporulation under two different starvation conditions. Cells were treated and spores enumerated as described in Fig 2B. Scale bars: TPM agar 500 μm, submerged culture 100 μm. (C) Immunoblot detection of DmxB in total cell extracts. Total cell lysates from cells of the indicated genotypes were harvested from starvation agar at 24 hrs of development, separated by SDS-PAGE and probed with rabbit, polyclonal α-DmxB serum. Protein from the same calculated number of cells was loaded per lane. DmxB has a calculated molecular mass of 35.3 kDa. Molecular mass marker is indicated on the left. The non-specific band above the band corresponding to DmxB serves as an internal loading control.

Mutations in two genes caused developmental defects without affecting growth or motility in vegetative cells [44]. MXAN3735, henceforth DmxB (DGC from *M*. *x**anthus* B), is a predicted cytoplasmic protein with an N-terminal receiver domain of two component system and a C-terminal GGDEF domain that contains the conserved residues for catalytic activity (G^219^GDEF) and an intact I-site (R^210^ESD), which allows c-di-GMP binding and allosteric feedback inhibition of DGC activity (Fig 3A; [44]). A mutant lacking DmxB neither aggregated on TPM agar nor in submerged culture and was strongly reduced in sporulation (S1 Fig and Fig 3B). MXAN2061, henceforth PmxA (PDE from *M*. *x*anthus A), is a predicted integral membrane protein and contains an N-terminal periplasmic Cache domain followed by a transmembrane segment, a HAMP domain and a HD-GYP domain with all the residues required for catalytic activity (H^424^D-G^485^YP) (Fig 3A; [44]). The Δ*pmxA* mutant formed highly irregular translucent fruiting bodies on TPM agar, did not aggregate in submerged culture (S1 Fig and Fig 3B) and only sporulated at 15% of WT levels (S1 Fig and Fig 3B). Moreover, the Δ*dmxB* Δ*pmxA* double mutant had the same developmental phenotype as the Δ*dmxB* strain (Fig 3B).

The developmental defects in all four mutants were complemented by ectopic expression of the relevant WT gene from its native promoter on plasmids integrated at the Mx8 *attB* site (Fig 3B and [15]). Because SgmT and TmoK are important for T4P-dependent motility, we speculate that the developmental defects caused by lack of either of these two proteins may be caused by a defect in T4P-dependent motility. From here on, we focused on DmxB and PmxA that are only important for development.

### DmxB and PmxA have enzymatic activity and DmxB binds c-di-GMP *in vitro*

**Fig 4:**
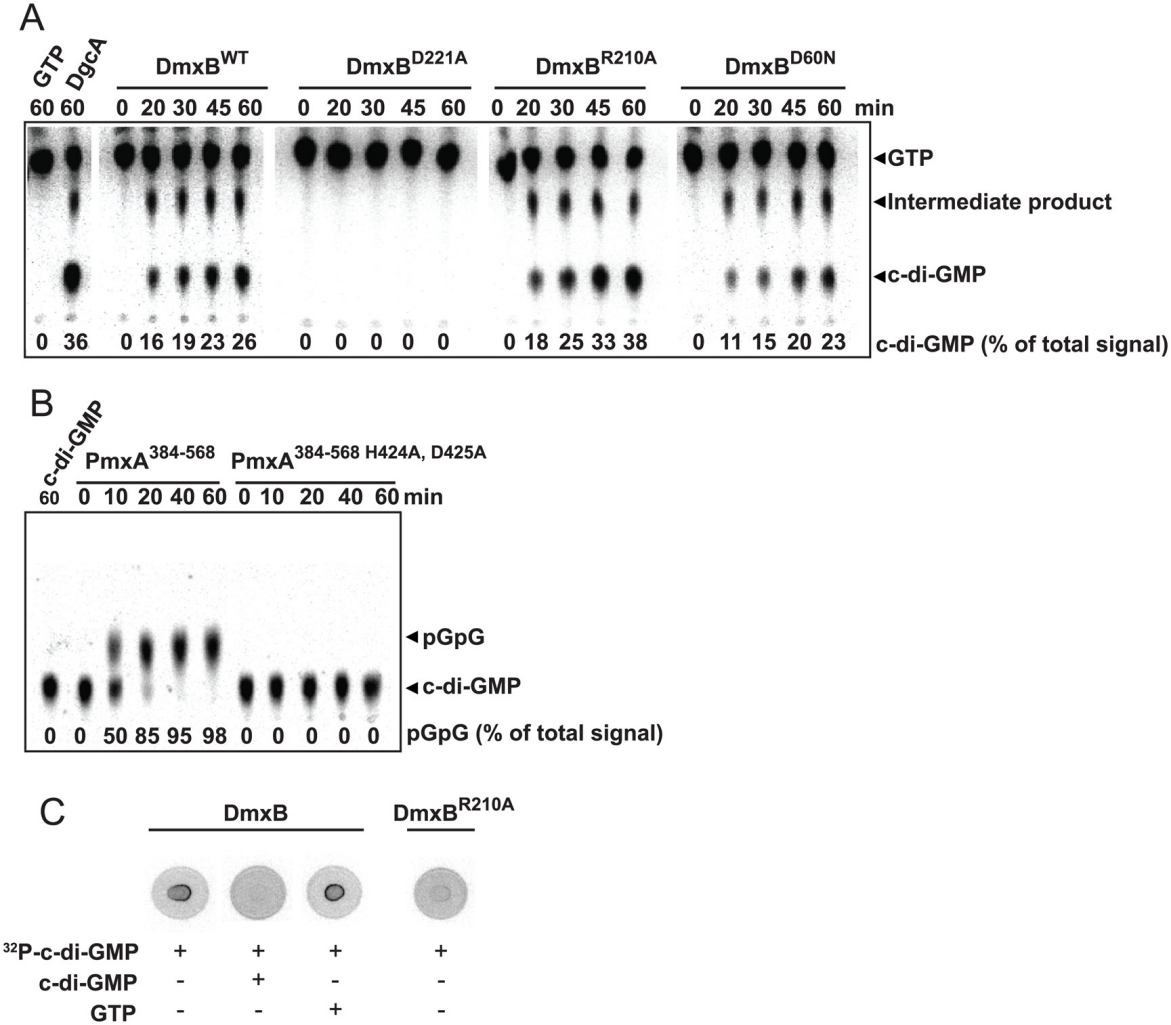
*In vitro* assay for enzyme activity and c-di-GMP binding. (A) DGC assay of DmxB variants. The indicated His6-tagged full-length DmxB variants were incubated with [α-^32^P]-GTP for the indicated periods of time followed by separation of nucleotides by TLC. Full-length DgcA^WT^ was used as a positive control. GTP and c-di-GMP are indicated. The intermediate product indicated was described as a product formed during the DGC-dependent synthesis of c-di-GMP [49]. Numbers at the bottom indicate levels of c-di-GMP in % of the total signal in each lane. (B) PDE activity assay of PmxA. The indicated PmxA variants were incubated with [α-^32^P]-labeled c-di-GMP for the indicated periods of time followed by separation of nucleotides by TLC. pGpG and c-di-GMP are indicated. Numbers at the bottom indicate levels of pGpG in % of the total signal in each lane. (C) DRaCALA to detect c-di-GMP binding. The indicated full-length DmxB variants were incubated with [α-^32^P]-c-di-GMP with or without unlabeled c-di-GMP or GTP as competitors as indicated. 10 μl of the reaction mixtures were transferred to a nitrocellulose filter, dried and imaged.

To test *in vitro* for enzymatic activity of DmxB and PmxA, we overexpressed His_6_-tagged full-length variants of DmxB and truncated variants of PmxA (Fig 4A and 4B) in *Escherichia coli* and purified them as soluble proteins.

Similarly to the control protein DgcA^WT^, DmxB produced c-di-GMP when incubated with [α-^32^P]-GTP as detected after separation of nucleotides by thin layer chromatography (TLC), while an active site variant DmxB^D221A^ did not (Fig 4A). To test whether DmxB binds c-di-GMP *in vitro* we used a differential radial capillary action of ligand assay (DRaCALA) with [α-^32^P]-labeled c-di-GMP [48]. In agreement with the predictions from sequence analyses, DmxB specifically bound [α-^32^P]-c-di-GMP whereas the I-site mutant DmxB^R210A^ did not (Fig 4C). Moreover, the I-site mutant had slightly increased DGC activity *in vitro* in comparison to the WT protein consistent with impaired feedback inhibition (Fig 4A). PmxA^384-568^, which contains the predicted cytoplasmic part of PmxA, displayed PDE activity and degraded [α-^32^P]-labeled c-di-GMP to linear pGpG, whereas the active site variant PmxA^H424A, D425A^ did not (Fig 4B).

### Lack of DmxB but not PmxA causes changes in the c-di-GMP level during starvation

**Fig 5:**
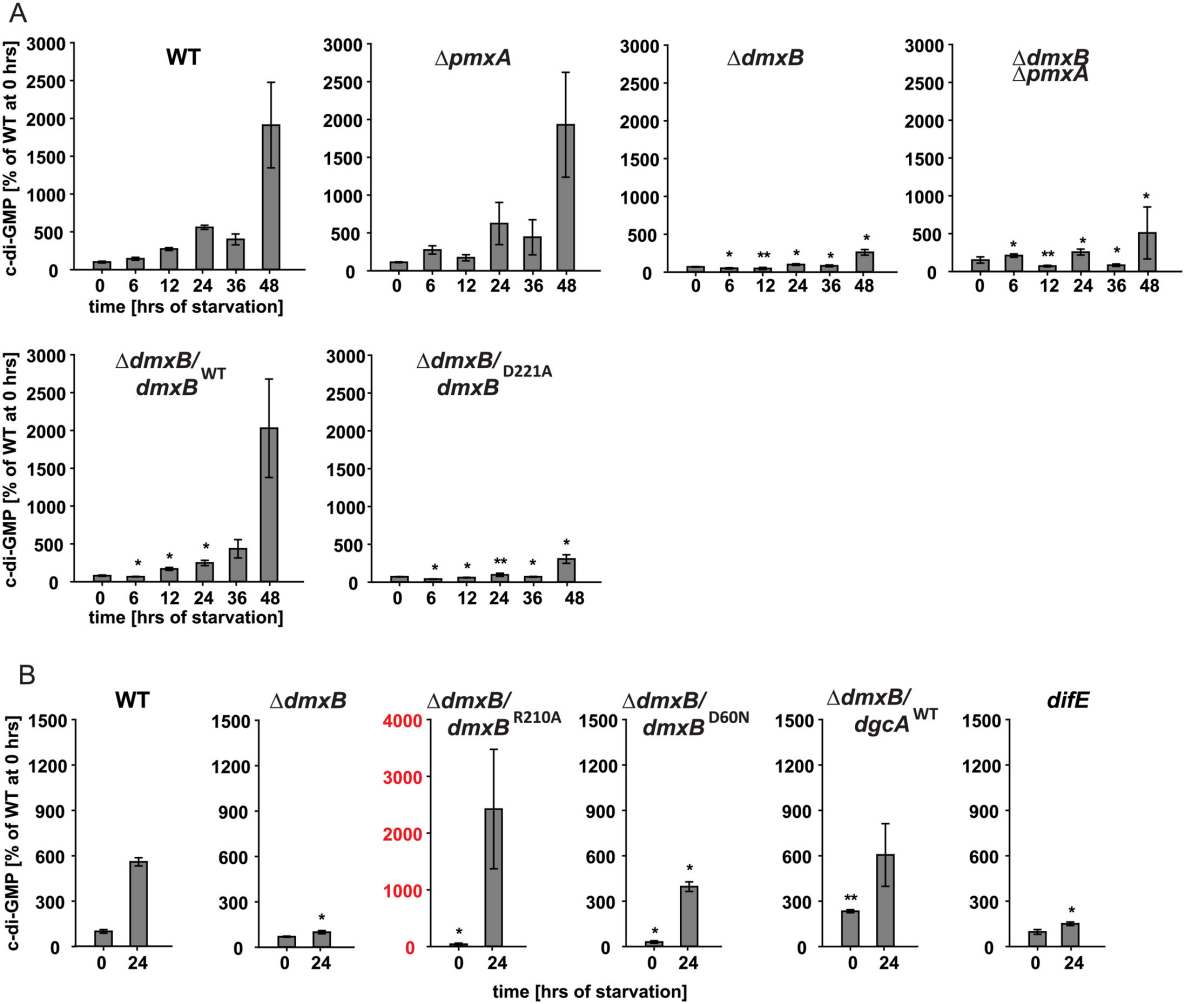
c-di-GMP levels in cells of the indicated mutants during starvation. (A, B) c-di-GMP levels in cells of the indicated genotypes were determined from three biological replicates as described in Fig 1. Note that the data for the WT are the same as in Fig 1 and in (B) the data for the WT and Δ*dmxB* strains are the same as in (A) and are shown again for the indicated time points for comparison. Note the different scale in (B) and for the Δ*dmxB/dmxB*^R210A^ strain. * p < 0.05, ** p < 0.001 in Student’s t-test comparing the different mutants to the WT at the same time points.

To determine if lack of DmxB or PmxA had an effect on c-di-GMP levels *in vivo*, we determined the c-di-GMP level in the Δ*dmxB and* Δ*pmxA* mutants starved in suspension for 48 hrs. In the Δ*pmxA* mutant, the c-di-GMP level in vegetative cells as well as during starvation was similar to that in WT (Fig 5A). In the Δ*dmxB* mutant, the c-di-GMP level in vegetative cells was also similar to that in WT and essentially remained constant throughout the entire time course without showing the ~20-fold increase observed in WT (Fig 5A). Moreover, the Δ*dmxB* Δ*pmxA* double mutant accumulated c-di-GMP at a similar low level as the Δ*dmxB* mutant (Fig 5A) consistent with the observation that the double mutant has the same developmental phenotype as the Δ*dmxB* mutant. Because the Δ*pmxA* mutant did not show significant changes in c-di-GMP levels during development and the Δ*dmxB* Δ*pmxA* double mutant accumulated c-di-GMP at a similar low level as the Δ*dmxB* mutant, we focused on elucidating the function of DmxB in development.

In the Δ*dmxB/dmxB*^WT^ complementation strain but not in the Δ*dmxB/dmxB*^D221A^ strain containing the active site variant of DmxB, the c-di-GMP level during starvation was restored to that in WT (Fig 5A). Importantly, the Δ*dmxB/dmxB*^D221A^ strain phenocopied the Δ*dmxB* mutant and did not aggregate and was strongly reduced in sporulation (Fig 3B). Moreover, development of the strain Δ*dmxB/dmxB*^R210A^, which contains the DmxB variant with a substitution of the conserved Arg residue in the I-site, proceeded as in WT. This strain had a c-di-GMP level that was ~4-fold higher than in WT at 24 hrs of starvation (Fig 5B—third panel, note scale on y-axis) consistent with the increased DGC activity *in vitro* (Fig 4A) and the notion that DmxB^R210A^ is no longer subject to feedback inhibition by c-di-GMP. In all three complementation strains, the DmxB variants had accumulated at the same level at 24 hrs and this level was lower than in the DK1622 WT (Fig 3C; Cf. below). We conclude that complementation of the Δ*dmxB* mutant depends on DGC activity by DmxB and not only on its presence, that DmxB is responsible for the ~20-fold increase in the c-di-GMP level during development and that this increase is essential for development whereas an even higher increase in c-di-GMP levels does not interfere with development.

Because DmxB contains an N-terminal receiver domain with the conserved phosphorylatable Asp residue conserved (D60), we asked if DmxB phosphorylation is involved in regulating DmxB activity. To this end, we ectopically expressed *dmxB*^D60N^, which encodes a DmxB variant in which this Asp residue has been substituted with non-phosphorylatable Asn. DmxB^D60N^ accumulated similarly to DmxB^WT^ in the Δ*dmxB*/*dmxB*^WT^ complementation strain (Fig 3C), complemented the developmental defects in the Δ*dmxB* mutant (Fig 3B) and largely restored c-di-GMP accumulation (Fig 5B). *In vitro* DmxB^D60N^ displayed DGC activity similar to DmxB^WT^ (Fig 4A). Altogether, these observations suggest that phosphorylation of the N-terminal receiver domain in DmxB is not essential for DmxB function. Of note, MXAN3734 located downstream of *dmxB* encodes a response regulator; however, under the conditions tested, this protein is not required for development (S3 Fig).

### Δ*dmxB* mutant is partially complemented by expression of a heterologous DGC

Strains that accumulate significantly more c-di-GMP than WT (WT/DgcA^WT^ and Δ*dmxB*/*dmxB*^R210A^) developed whereas the strains (WT/PA5295^WT^, Δ*dmxB*, and Δ*dmxB*/*dmxB*^D221A^) that accumulate significantly less c-di-GMP did not, suggesting that a minimal threshold level of c-di-GMP is essential for development and that significantly higher c-di-GMP levels do not interfere with development. Because DmxB is responsible for reaching this threshold, this raised the question if the only function of DmxB is to contribute to the cellular pool of c-di-GMP. To address this question, we expressed the heterologous DgcA^WT^ or its active site variant DgcA^D164A^ in the Δ*dmxB* mutant. Interestingly, fruiting body formation and sporulation were largely restored by expression of DgcA^WT^ but not by DgcA^D164A^ (Fig 3B) and in the DgcA^WT^ containing strain the level of c-di-GMP was similar to that in WT at 24 hrs of starvation (Fig 5B) suggesting that the major function of DmxB is to contribute to a cellular pool of c-di-GMP in developing *M*. *xanthus* cells without engaging in specific protein-protein interactions.

### DmxB specifically accumulates during development

**Fig 6:**
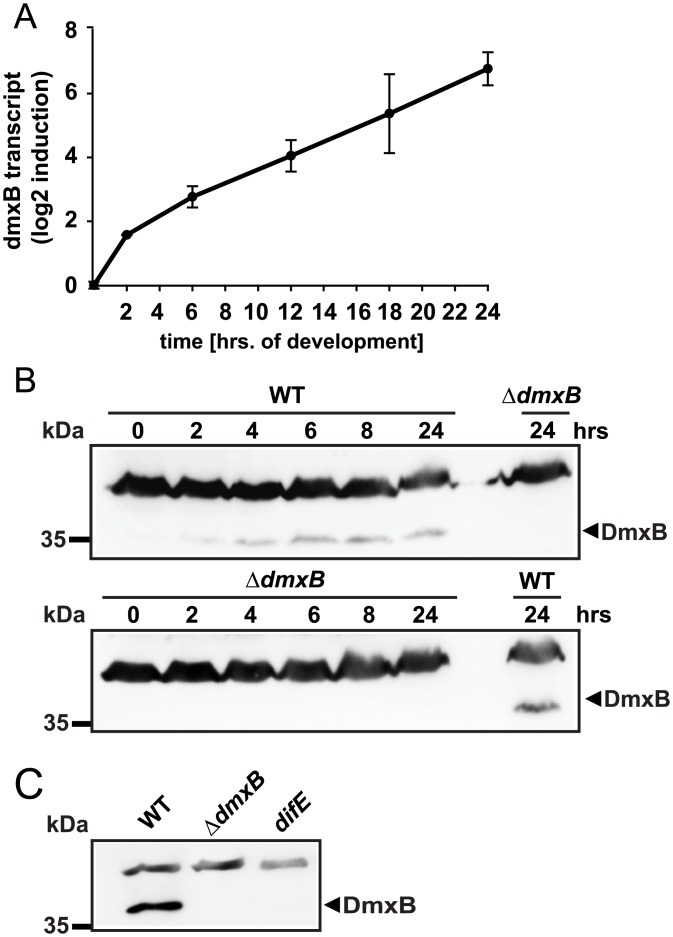
Determination of *dmxB* transcript and DmxB accumulation levels. (A) qRT-PCR analysis of *dmxB* expression. Total RNA was isolated from WT developed in submerged culture at the indicated time points. *dmxB* transcript level is shown as the mean ± standard deviation from two biological replicates with each three technical replicates relative to WT at 0 hrs. (B) Immunoblot detection of DmxB in total cell extracts. Total cell lysates were prepared from cells of the indicated genotypes harvested from starvation agar plates at the indicated time points of development. Immunoblots were prepared as described in Fig 3C. Protein from the same calculated number of cells was loaded per lane. Molecular mass marker is indicated on the left. The non-specific band above the band corresponding to DmxB serves as an internal loading control. (C) Immunoblot detection of DmxB in total cell extract of WT, Δ*dmxB* and *difE* strains. Total cell lysates from cells harvested from starvation agar after 24 hrs of development were prepared. Immunoblots were prepared as described in Fig 3C. Protein from the same calculated number of cells was loaded per lane. Molecular mass marker is indicated on the left. The non-specific band above the band corresponding to DmxB serves as an internal loading control.

To deduce how lack of DmxB only causes developmental defects and not a defect in T4P-dependent motility in growing cells, we determined the expression pattern of *dmxB* using qRT-PCR. The *dmxB* transcript level increased >100-fold during the first 24 hrs of development in comparison to growing cells (Fig 6A). Also, immunoblot analysis revealed that DmxB was undetectable in growing cells and that accumulation increased during development (Fig 6B). Together, these data demonstrate that DmxB accumulation is regulated at the transcriptional level and induced in response to starvation.

### Lack of DmxB causes reduced EPS accumulation

**Fig 7:**
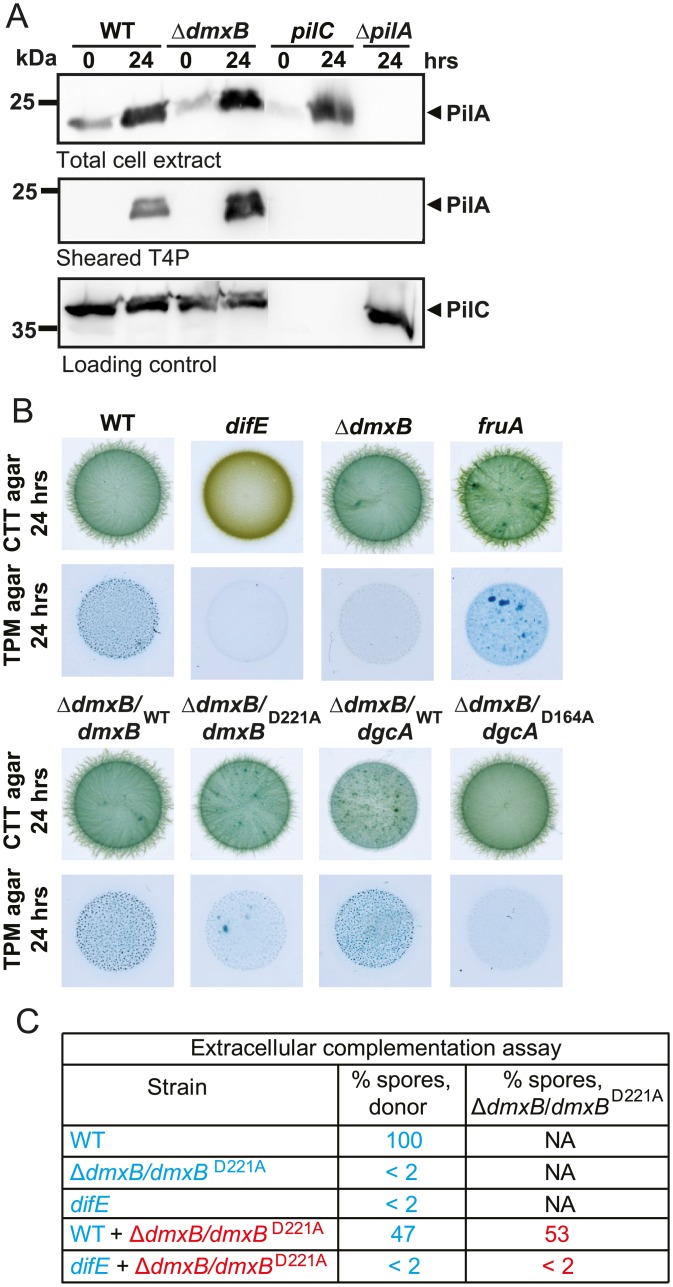
T4P formation and EPS accumulation in various mutants. (A) Immunoblot detection of PilA in total cell extracts and in the sheared T4P fraction. In the upper and lower blots, total cell extracts were isolated from the indicated strains developed in submerged culture at the indicated time points. In the middle blot, T4P were sheared off from the same number of cells and concentrated by MgCl2 precipitation. In all three blots, protein from the same calculated number of cells was loaded per lane. The upper and middle blots were probed with α-PilA antibodies. The lower blot was probed against PilC, which is important for T4P assembly and served as a loading control. PilA and PilC have a calculated molecular mass 23.4 kDa and 45.2 kDa, respectively. Molecular mass marker is indicated on the left. (B) Quantification of EPS accumulation in *dmxB* mutants. 20 μl aliquots of exponentially growing cells of the indicated genotypes were spotted at a density of 7 × 10^9^ cells/ml on 1.5% agar supplemented with 0.5% CTT and 20 μg/ml trypan blue or on TPM starvation agar supplemented with 20 μg/ml trypan blue and incubated at 32°C for 24 hrs. (C) Extracellular complementation assay of Δ*dmxB* mutant. Cells of the indicated genotypes were either developed alone as described in Fig 2B in submerged culture or mixed at a 1:1 ratio and co-developed in submerged culture. Sporulation levels are enumerated after 120 hrs of starvation as the number of germinating heat- and sonication resistant spores relative to WT (100%).

In growing cells c-di-GMP is important for T4P-dependent motility by regulating T4P formation and EPS accumulation. To elucidate the mechanism underlying the developmental defects of the Δ*dmxB* mutant, we therefore tested this mutant for PilA accumulation and T4P formation. In WT cells as well as in the Δ*dmxB* mutant the level of PilA in total cell extracts increased from 0 to 24 hrs of development as previously reported for WT [50] (Fig 7A). Also, the level of PilA incorporated into T4P increased significantly in both strains and even more in the Δ*dmxB* mutant than in the WT (Fig 7A). As expected, PilA was not detected in the Δ*pilA* mutant and also not in T4P fraction of the *pilC* mutant that served as negative controls (Fig 7A).

EPS accumulation was determined using an assay in which trypan blue binding to EPS is used to visualize EPS. For this purpose, cells were inoculated on solid medium containing trypan blue in the presence or absence of nutrients. As expected, in the presence of nutrients, no differences in trypan blue staining were observed between WT, a *fruA* mutant, which has a developmental defect [51], the Δ*dmxB* mutant and the Δ*dmxB* mutant complemented with *dmxB*^WT^, *dmxB*^D221A^, *dgcA*^WT^ or *dgcA*^D164A^ whereas the *difE* mutant, which lacks the histidine protein kinase DifE of the Dif chemosensory system that is essential for EPS accumulation, did not stain with trypan blue (Fig 7B). By contrast, in the absence of nutrients, only the WT, Δ*dmxB*/*dmxB*^WT^ and Δ*dmxB*/*dgcA*^WT^ strains accumulated high levels of EPS as indicated by the dark blue coloration, while the Δ*dmxB*, Δ*dmxB*/*dmxB*^D221A^ and Δ*dmxB*/*dgcA*^D164A^ strains, similarly to the *difE* strain, bound trypan blue at a much reduced level (Fig 7B). Importantly, the development-deficient *fruA* mutant bound trypan blue similarly to WT providing evidence that the reduced EPS accumulation in the Δ*dmxB*, Δ*dmxB*/*dmxB*^D221A^ and Δ*dmxB*/*dgcA*^D164A^ strains was not a simple consequence of lack of development. Together, these data strongly indicate that an increase in the c-di-GMP level is required for EPS accumulation during development and that DmxB is responsible for this increase in WT.

### Lack of DmxB causes non-cell autonomous developmental defects

The developmental defects of the Δ*dmxB* mutant are similar to those of the *difE* mutant, i.e. no aggregation, strongly reduced sporulation and strongly reduced EPS accumulation. Therefore, we hypothesized that *difE* would also be important for DmxB accumulation. As shown in Fig 6C, the *difE* mutant is strongly reduced in DmxB accumulation. Consistently, the *difE* mutant was found to be strongly reduced in c-di-GMP accumulation after 24 hrs of starvation (Fig 5B).

Because the developmental defects of a *difE* mutant can be rescued by extracellular complementation by WT in co-development assays, we reasoned that the Δ*dmxB* mutant would also be rescued by co-development with WT if the primary defect in this mutant is reduced EPS accumulation during development. To this end, cells of the tetracycline resistant Δ*dmxB* mutant (Δ*dmxB/dmxB*^D221A^) were mixed with tetracycline sensitive WT cells in a 1:1 ratio and co-developed in submerged culture. Subsequently, spores formed by the two strains were enumerated. In this experiment, 53% of germinating spores derived from the Δ*dmxB/dmxB*^D221A^ strain (Fig 7C). Importantly, the Δ*dmxB* mutant was not rescued by co-development with the *difE* mutant and the Δ*dmxB* mutant did not rescue sporulation of the *difE* mutant whereas the WT efficiently rescued sporulation by the *difE* mutant, supporting the notion that the mechanism underlying the Δ*dmxB* developmental defects is indeed reduced EPS accumulation.

*Dictyostelium discoideum* is the only eukaryote where c-di-GMP has been identified and lack of the DGC DgcA blocks fruiting body formation [52]. Development of a *dgcA* mutant is restored by exogenous c-di-GMP at a final concentration of 1 mM. Therefore, we tested if exogenous c-di-GMP would restore development of the Δ*dmxB* mutant. Estimates of the intracellular concentration of c-di-GMP in different bacterial species range between 130 nM to a few μM [53, 54]. Therefore, we added exogenous c-di-GMP at 0 or 24 hrs to a final concentration of 1 mM to the Δ*dmxB* mutant in submerged culture. However, development of the mutant was not restored by exogenous c-di-GMP under these conditions.

### Transcription of *eps* genes is reduced in the Δ*dmxB* mutant

**Fig 8:**
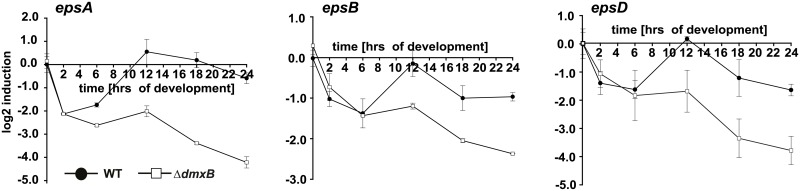
c-di-GMP regulates *epsABD* transcription. Total RNA was isolated at the indicated time points from cells of WT (closed circles) and the Δ*dmxB* mutant (open squares) developed in submerged culture. Transcript levels are shown as mean ± standard deviation from two biological replicates with each three technical replicates relative to WT at 0 hrs.

The *eps* locus encodes proteins involved in EPS synthesis and transport and at least 10 of the genes in this locus are essential for development [41] (S4A Fig). We measured the expression profile in WT and the Δ*dmxB* mutant during development of ten of the *eps* genes, which encode proteins with different functions in EPS synthesis and transport using qRT-PCR (Fig 8 and S5AB Fig). For seven of the ten genes we did not observe significant differences in the expression profiles between the two strains (S4B Fig); however, three genes (*epsA*, *epsB* and *epsD*) were transcribed at a significantly lower level in the Δ*dmxB* mutant than in WT at the late time points (Fig 8). These three genes have been suggested to form a single transcriptional unit together with two additional genes [41] (S4A Fig). *epsA* and *epsD* encode predicted glycosyltransferases and have been shown to be essential for EPS accumulation and development while e*psB* encodes a predicted glycosyl hydrolase that is neither important for EPS accumulation nor for development [41].

**Fig 9:**
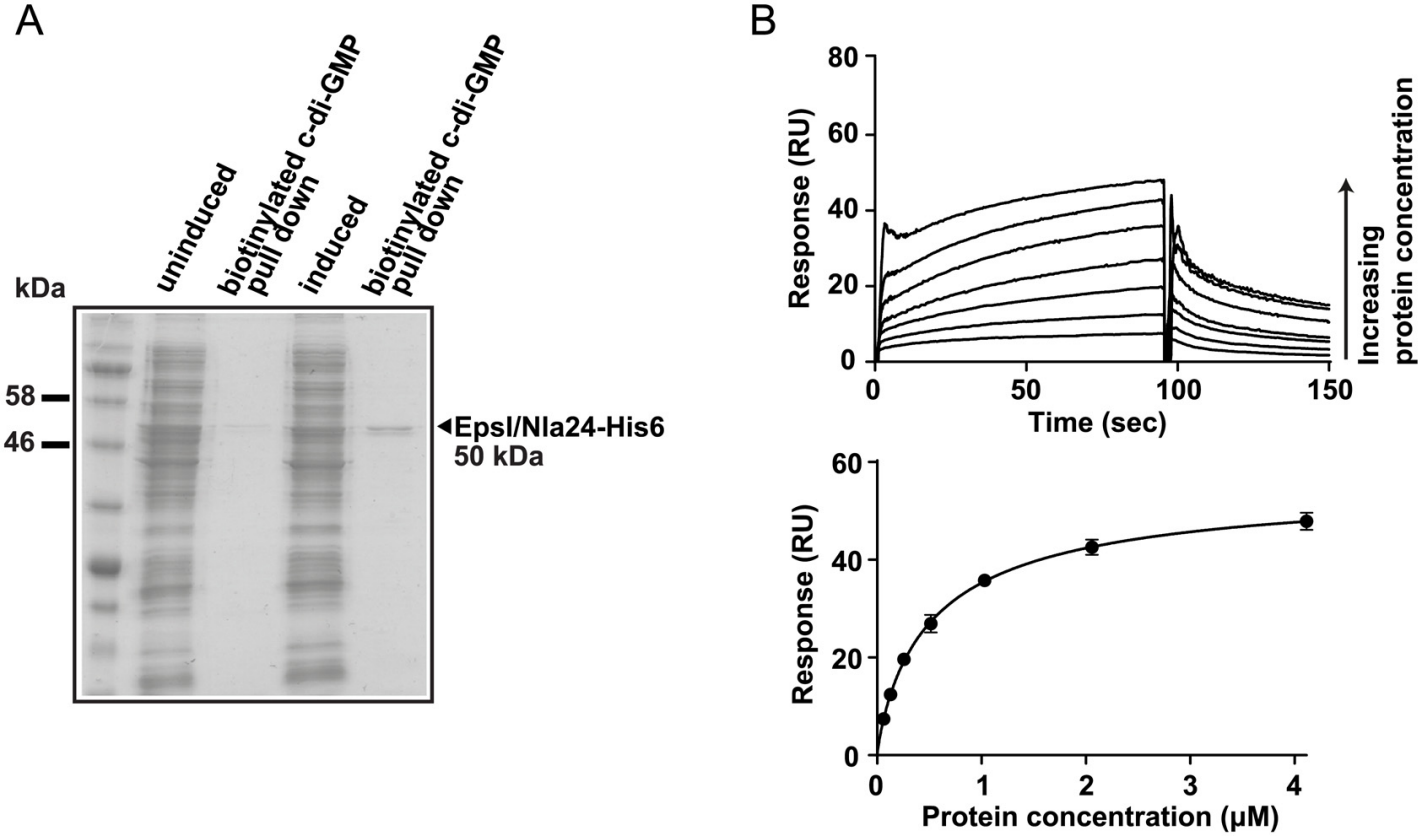
Transcriptional regulator EpsI/Nla24 binds c-di-GMP. (A) Pull-down experiment with the soluble fraction of *E*. *coli* cell lysates before and after induction of EpsI/Nla24 synthesis using biotinylated c-di-GMP immobilized on streptavidin magnetic beads. Cell extract from the uninduced sample was used as a negative control. Pulled-down protein is indicated with an arrow. EpsI/Nla24-His_6_ has a calculated molecular mass of 50 kDa. (B) SPR sensorgrams and resulting affinity fit data for EpsI/Nla24 binding to biotinylated c-di-GMP. Upper panel, sensorgrams of EpsI/Nla24 binding to biotinylated c-di-GMP immobilized on sensor chip. The concentration of EpsI/Nla24 ranged from 62.5 nM (lowest curve) to 4 μM (highest curve) and concentration replicates were included as appropriate. The protein binding and dissociation phases for all sensorgrams are shown. Lower panel, affinity fit of EpsI/Nla24 binding to biotinylated c-di-GMP. For this fit, binding responses were measured 4 sec before the end of the injection.

These data suggest that the DmxB-dependent high level of c-di-GMP that accumulates during development functions to stimulate transcription of at least three genes in the *eps* locus. The only transcription regulator known to be required for EPS synthesis in *M*. *xanthus* is the NtrC-like transcriptional regulator EpsI/Nla24 [41–43], which is encoded in the *eps* locus (S4A Fig). Two NtrC-like transcriptional regulators have been shown to bind to c-di-GMP [22, 23], suggesting a possible route for c-di-GMP regulation of EPS synthesis via direct allosteric control of EpsI/Nla24. To test whether EpsI/Nla24 binds to c-di-GMP, we first used a biotinylated c-di-GMP pull-down experiment. As shown in Fig 9A, EpsI/Nla24-His_6_ was successfully pulled-down from *E*. *coli* whole-cell extracts containing overexpressed EpsI/Nla24-His_6_, strongly suggesting c-di-GMP binding. Direct c-di-GMP binding by EpsI/Nla24 was subsequently confirmed using Surface Plasmon Resonance (SPR) with a chip containing biotinylated c-di-GMP bound to streptavidin, and the purified protein (Fig 9B) [55]. The K_D_ of c-di-GMP binding to EpsI/Nla24 was calculated as 0.53 ±0.06 μM, well within the physiological range of published c-di-GMP binding proteins (Fig 9B) [56].

## Discussion

Here, we show that c-di-GMP is an essential regulator of starvation-induced development with fruiting body formation and sporulation in *M*. *xanthus*. To assess c-di-GMP accumulation during starvation, *M*. *xanthus* cells were starved on a solid surface or in suspension. While starvation in suspension is not conducive to development, starvation on a solid surface is. Under both conditions, the c-di-GMP level increased significantly (~20-fold over 48 hrs). Because the level of c-di-GMP does not increase significantly in stationary phase *M*. *xanthus* cells [44], we conclude that the increase in the c-di-GMP level is a specific response to starvation and that the c-di-GMP level increases during development. In otherwise WT cells, further increasing the c-di-GMP level by expression of a heterologous DGC did not prevent progression of development whereas reducing the c-di-GMP level by expression of a heterologous PDE caused defects in fruiting body formation as well as in sporulation suggesting that a threshold level of c-di-GMP is essential for development to proceed to completion.

By systematically analyzing a set of mutants with single mutations in the 24 gene encoding proteins with a GGDEF, EAL or HD-GYP domain, we identified a single catalytically active DGC, DmxB, which is not only specifically required for development but also responsible for the increase in the c-di-GMP level during starvation. DGC activity by DmxB is essential for development. Moreover, the DmxB-dependent increase in the c-di-GMP level during development is necessary for EPS accumulation and our data suggests that the stimulation of EPS accumulation proceeds via stimulation of the transcription of a subset of the genes in the *eps* locus. This subset of genes code for predicted glycosyltransferases that have previously been shown to be important for EPS accumulation and development (EpsA, EpsD) and a glycosyl hydrolase (EpsB) that is neither important for EPS accumulation nor for development [41]. Also, the developmental defects caused by lack of DmxB are non-cell autonomous and development of the Δ*dmxB* mutant can be rescued by co-development with a strain proficient in EPS accumulation strongly suggesting that the defects in development in the Δ*dmxB* mutant are caused by lack of EPS. DmxB can be largely functionally replaced by a heterologous DGC both with respect to development and EPS accumulation. Because it is unlikely that this DGC would be able to engage in the same protein-protein interactions as DmxB, we infer that the increase in c-di-GMP level, rather than DmxB *per se*, is important for development and EPS accumulation. Finally, because all strains with significant increases in the level of c-di-GMP, irrespective of the DGC involved, develop whereas strains with reduced c-di-GMP levels have strong defects in development, we surmise that a minimal threshold level of c-di-GMP is essential for development to be successfully completed and that c-di-GMP levels in excess of this threshold do not interfere with development. In WT cells, this minimal threshold level of c-di-GMP is generated by DmxB. It should be noted that simply increasing the c-di-GMP level in vegetative cells to that observed during development by expression of a heterologous DGC is not sufficient to initiate fruiting body formation [44]. Thus, as opposed to the second messenger (p)ppGpp, which is required and sufficient for initiating the developmental program in *M*. *xanthus* [57, 58], the increased c-di-GMP level is necessary for development but not sufficient to initiate this program. In *S*. *venezuelae* c-di-GMP also regulates multicellular development with the formation of aerial hyphae. However, in this organism, a high level of c-di-GMP inhibits development by binding to the transcription factor BldD, which inhibits expression of sporulation genes, and a decrease in the c-di-GMP level stimulates development [5]. Thus, c-di-GMP has opposite effects on multicellular development in *S*. *venezuelae* and *M*. *xanthus*.

EPS synthesis is a target of c-di-GMP-dependent regulation in several bacterial species [for review, see [1]]. This regulation can occur at the transcriptional and post-translational level. Among transcription factors regulating the expression of genes for EPS synthesis, c-di-GMP has been shown to bind to and modulate the activity of the NtrC-like transcriptional regulators FleQ in *P*. *aeruginosa* [22] and VpsR in *Vibrio cholerae* [23] as well as the response regulator VpsT in *V*. *cholerae* [24]. FleQ alone inhibits transcription of the *pel* operon involved in EPS synthesis while binding of c-di-GMP to FleQ inhibits its binding to the *pel* promoter in that way causing derepression of *pel* transcription [22]. VpsR binds to its cognate promoters independently of c-di-GMP; however, it only functions as a transcriptional activator in the c-di-GMP bound state and it is currently not known how c-di-GMP modulates VpsR activity [23]. The NtrC-like transcriptional regulator EpsI/Nla24 is the only transcription regulator known to be required for EPS synthesis in *M*. *xanthus* and is composed of three domains, an N-terminal receiver domain of two component system, a AAA+ domain, and a helix-turn-helix DNA-binding domain (S5 Fig). As shown here, EpsI/Nla24 binds tightly to c-di-GMP, with a K_D_ in the low μM range. This strongly suggests that regulation of EPS synthesis during development by c-di-GMP in *M*. *xanthus* proceeds through control of transcription factor activity, and hence *eps* transcription, by direct c-di-GMP binding to EpsI/Nla24. Recently, structural insights into how the AAA+ domain in FleQ from *P*. *aeruginosa* binds c-di-GMP were reported and several important motifs for c-di-GMP binding were identified [59] (S5 Fig). Alignment of the AAA+ domains of EpsI/Nla24 and FleQ revealed that not all of these motifs are present in EpsI/Nla24 but confirmed the presence of two Arg residues in EpsI/Nla24 (S5 Fig) that are important for c-di-GMP binding by FleQ [59]. Similarly, VpsR from *V*. *cholera* does not have all the binding residues reported for FleQ [59] but still binds c-di-GMP [23].

EpsI/Nla24 has been suggested to regulate *eps* gene transcription not only in developing cells but also during vegetative growth [43]. To explain the effect of lack of DmxB and by implication low c-di-GMP levels on *eps* gene expression during development, we suggest three scenarios. First, as the level of c-di-GMP in vegetative WT cells is similar to the c-di-GMP level in starving Δ*dmxB* cells, it is possible that EpsI/Nla24 binds c-di-GMP at this level in vegetative cells as well as in starving Δ*dmxB* cells. At this c-di-GMP level, EpsI/Nla24 in complex with c-di-GMP activates transcription of all the tested *eps* genes in vegetative cells and a subset of the tested *eps* genes in developing cells; however, a higher level of c-di-GMP is required for *epsABD* expression during development. Alternatively, EpsI/Nla24 functions independently of c-di-GMP in vegetative cells and only binds c-di-GMP during development and this binding requires the high concentration of c-di-GMP that is observed in starving WT cells. In this scenario, EpsI/Nla24 in complex with c-di-GMP specifically functions to activate *epsABD* expression. Of note, c-di-GMP modulates FleQ activity at different promoters differentially [60]. In a third scenario, an additional transcriptional regulator could be involved in the response to the high c-di-GMP level during development. In future experiments, the molecular mechanism of EpsI/Nla24 in the expression of *eps* genes will be analyzed.

Several lines of evidence suggest that synthesis and activity of DmxB is tightly regulated. First, DmxB only accumulates in starving cells but not in growing cells. Measurements of *dmxB* transcript levels strongly suggest that DmxB accumulation is regulated at the transcriptional level. DmxB consists of an N-terminal receiver domain and the C-terminal catalytically active GGDEF domain. An active DGC is an obligate dimer [8] and it was previously reported that DGCs can be induced to dimerize by phosphorylation of their receiver domain as in the case of PleD and WspR [61, 62]. However, the non-phosphorylatable variant DmxB^D60N^ was fully functional *in vivo* as well as *in vitro* suggesting that DmxB forms a dimer independently of phosphorylation of the N-terminal receiver domain. Second, a DmxB variant with a mutated I-site showed higher activity *in vitro* and accumulated ~4-fold more c-di-GMP than WT during development suggesting that DmxB is subject to allosteric feedback inhibition of DGC activity by c-di-GMP. The DmxB variant with the mutated I-site developed normally, suggesting that allosteric feedback inhibition of DGC activity by DmxB is not essential. We speculate that this feedback serves to minimize futile c-di-GMP synthesis during starvation. In total, these observations suggest that DmxB is regulated at the transcriptional level as well as post-translationally by allosteric feedback inhibition.

The Dif chemosensory system is essential for EPS synthesis in growing as well as in starving cells [40], however, it is not known how the Dif system stimulates EPS synthesis. Here, we show that the DifE histidine protein kinase is essential for DmxB accumulation and c-di-GMP accumulation during development, strongly suggesting that during development the Dif system functions by stimulating DmxB accumulation and in that way c-di-GMP and EPS synthesis. Because DmxB specifically accumulates during development and does not accumulate in growing cells, these data also argue that Dif functions through a different downstream target in growing cells to stimulate EPS synthesis.

In addition to DmxB, we also identified the enzymatically active PDE PmxA as specifically important for development. Interestingly, the Δ*pmxA* mutant did not show significant changes in the c-di-GMP levels during development suggesting that the developmental defects in the Δ*pmxA* mutant are not a simple consequence of changes in the global cellular c-di-GMP level but may involve protein-protein interaction and possibly also a local c-di-GMP pool. Interestingly, the HD-GYP domain protein RpfG from *Xanthomonas campestris* was found to interact directly with several GGDEF domain proteins. This interaction was independent on PDE activity of RpfG and DGC activity of the GGDEF domain proteins [63, 64]. It remains to be shown if PDE activity is essential for PmxA function *in vivo* and if PmxA interacts with other proteins involved in c-di-GMP metabolism in *M*. *xanthus*.

Proteins involved in c-di-GMP metabolism and regulation are ubiquitous with some species encoding >100 proteins with GGDEF, EAL, HD-GYP and effector domains [1]. Yet, mutation of individual genes can give rise to specific defects raising the question how these enzymes and effectors are regulated to obtain specific output responses. It has been suggested that individual signaling modules can be temporally separated by differentially regulating their synthesis, spatially separated by complex formation or by localizing to distinct subcellular locations, or by effectors having different binding affinities for c-di-GMP [1, 53, 54, 65]. Among the 17 GGDEF domain proteins in *M*. *xanthus*, 11 are predicted to have DGC activity [44]. We previously showed that DmxA has DGC activity and is involved in regulating EPS accumulation in growing *M*. *xanthus* cells. Lack of DmxA causes a slight but significant increase in the c-di-GMP level and a ~4-fold increase in EPS accumulation and in that way also cause a defect in T4P-dependent motility [44]. However, lack of DmxA does not cause developmental defects. Vice versa, lack of DmxB only causes developmental defects and not motility defects in growing cells. The finding here that DmxB is exclusively synthesized in developing cells provides evidence that *M*. *xanthus* restricts the synthesis of at least one DGC to a distinct stage of its life cycle suggesting that temporal regulation of proteins involved in c-di-GMP metabolism could be of general importance in *M*. *xanthus*. Similarly, it was recently demonstrated that *Bdellovibrio bacteriovorus* uses different DGCs at different stages of its predatory life cycle [66].

## Materials and Methods

### *M*. *xanthus* strains, growth and development

All *M*. *xanthus* strains are derivatives of the WT strain DK1622 [67]. *M*. *xanthus* strains and plasmids used in this work are listed in Tables 1 and 2, respectively. *M*. *xanthus* cells were grown in liquid 1% CTT medium or on 1% CTT agar plates at 32°C [68]. For development, cells were grown as described, harvested and resuspended in MC7 buffer (10 mM MOPS pH 7.0, 1 mM CaCl_2_) to a calculated density of 7 × 10^9^ cells/ml. 20 μl aliquots of cells were placed on TPM agar (10 mM Tris-HCl pH 7.6, 1 mM K_2_HPO_4_/KH_2_PO_4_ pH 7.6, 8 mM MgSO_4_); for development in submerged culture, 50 μl of the cell suspension were mixed with 350 μl MC7 buffer and placed in a 18 mm diameter microtiter dish. Cells were visualized at the indicated time points using a Leica MZ8 stereomicroscope or a Leica IMB/E inverted microscope and imaged using Leica DFC280 and DFC350FX CCD cameras, respectively. Sporulation levels were determined after development for 120 hrs in submerged culture as the number of sonication- and heat-resistant spores relative to WT [51]. In extracellular complementation experiments, spore titers were determined as the number of germinating spores relative to WT. Spores of mixed strains were enumerated by replica plating onto plates containing relevant antibiotics. Because the results of sporulation assay are highly variable, we considered it as significant only if the difference between strains were 3-fold or more. Kanamycin and oxytetracycline were added to *M*. *xanthus* cells at concentrations of 40 μg/ml or 10 μg/ml, respectively. Growth was measured as an increase in OD at 550 nm.

**Table 1:** *M*. *xanthus* strains used in this work.

*M*. *xanthus* strains	Genotype ^a^^,^ ^b^	Reference
DK1622	Wild-type	[67]
DK1300	*pilC*	[35]
SA3502	Δ*sgmT*	[15]
SW501	*difE*::kan^R^	[75]
DK10410	Δ*pilA*	[76]
DK11063	*fruA*::*Tn5 lacΩ7540*; kan^R^	[51]
SA3535	*attB*::pTP110; (P_*pilA*_-PA5295^WT^-strepII)	[44]
SA3537	*attB*::pTP112; (P_*pilA*_-PA5295^E328A^-strepII)	[44]
SA3543	*attB*::pTP114; (P_*pilA*_-*dgcA*^WT^-strepII)	[44]
SA3559	*attB*::pTP131; (P_*pilA*_-*dgcA*^D164A^-strepII)	[44]
SA3524	Δ*MXAN2424* (5–256/275)	[44]
SA3525	Δ*MXAN2530* (11-406/416)	[44]
SA3544	Δ*MXAN4232* (6–407/412)	[44]
SA3546	Δ*pmxA* (16–559/569)	[44]
SA3554	Δ*tmoK* (11–1101/1110)	[44]
SA3533	Δ*MXAN5791* (11–338/348)	[44]
SA3545	Δ*MXAN5199* (6–297/304)	[44]
SA3548	Δ*MXAN4675* (11–352/372)	[44]
SA3555	Δ*MXAN1525* (6–289/294)	[44]
SA3556	Δ*MXAN2643* (11–355/265)	[44]
SA3557	Δ*MXAN4029* (6–286/292)	[44]
SA3558	Δ*MXAN2807* (6–655/661)	[44]
SA3569	Δ*MXAN4257* (6–589/595)	[44]
SA3599	Δ*actA* (11-292/302)	[44]
SA5524	Δ*MXAN2997* (9–641/646)	[44]
SA5600	Δ*MXAN4463* (46–433/458)	[44]
SA5605	Δ*dmxB* (10–310/319)	[44]
SA5606	Δ*MXAN7362* (10–665/674)	[44]
SA5607	Δ*MXAN5366* (10–312/321)	[44]
SA5525	Δ*MXAN5340* (50–542/547)	[44]
SA5526	Δ*MXAN5053* (6–612/617)	[44]
SA5527	Δ*MXAN6098* (6–495/501)	[44]
SA5619	Δ*dmxB*; *attB*::pDJS27 (P_nat_-*dmxB*^WT^)	This study
SA5620	Δ*dmxB*; *attB*::pDJS37 (P_nat_-*dmxB*^D221A^)	This study
SA5621	Δ*dmxB*; *attB*::pDJS33 (P_nat_-*dmxB*^D60N^)	This study
SA5622	Δ*dmxB*; *attB*::pDJS38 (P_nat_-*dmxB*^R210A^)	This study
SA5636	Δ*dmxB*; *attB*::pTP114 (P_*pilA*_-*dgcA*^WT^-strepII)	This study
SA5637	Δ*dmxB*; *attB*::pTP131 (P_*pilA*_-*dgcA*^D164A^-strepII)	This study
SA5629	Δ*pmxA*; *attB*::pDJS56 (P_nat_-*pmxA*^WT^)	This study
SA5630	Δ*tmoK*; *attB*::pDJS57 (P_nat_-*tmoK*^WT^)	This study

^a^ For in-frame deletions, numbers in brackets indicate the codons deleted over the total number of codons in a given gene).

^b^ For strains containing plasmids integrated at the Mx8 *attB* site, the gene expressed including the promoter driving the expression is indicated in brackets.

**Table 2:** Plasmids used in this work.

Plasmids	Description	Reference
pBJ114	kan^R^, *galK*	[77]
pSWU30	tet^R^	[50]
pSW105	P_*pilA*_, kan^R^	[78]
pET24b(+)	kan^R^, expression vector	Novagen
pMALc2x	amp^R^, expression vector	New England Biolabs
pDJS27	pSWU30; P_nat_-*dmxB*^WT^; tet^R^	This study
pDJS37	pSWU30; P_nat_-*dmxB*^D221A^; tet^R^	This study
pDJS33	pSWU30; P_nat_-*dmxB*^D60N^; tet^R^	This study
pDJS38	pSWU30; P_nat_-*dmxB*^R210A^; tet^R^	This study
pDJS56	pSWU30; P_nat_-*pmxA*; tet^R^	This study
pDJS57	pSWU30; P_nat_-*tmoK*; tet^R^	This study
pDJS31	pET24b(+); *dgcA*^WT^; kan^R^	[44]
pDJS30	pET24b(+); *dmxB*^WT^; kan^R^	This study
pDJS39	pET24b(+); *dmxB*^D221A^; kan^R^	This study
pDJS42	pET24b(+); *dmxB*^R210A^; kan^R^	This study
pDJS45	pET24b(+); *dmxB*^D60N^; kan^R^	This study
pDJS71	pET24b(+); *pmxA*^384-568^; kan^R^	This study
pDJS75	pET24b(+); *pmxA*^384-568 H424A, D425A^; kan^R^	This study
pDJS79	pET24b(+); *epsI*/*nla24*; kan^R^	This study
pTP114	pSW105, P_*pilA*_-*dgcA*^WT^-strepII, kan^R^	[44]
pTP131	pSW105, P_*pilA*_-*dgcA*^D164A^-strepII, kan^R^	[44]

*E*. *coli* strains were grown in LB broth in the presence of relevant antibiotics [69]. All plasmids were propagated in *E*. *coli* Mach1 (Δ*recA*1398 *endA*1 *tonA* Φ80Δ*lacM*15 Δ*lacX*74 *hsdR*(r_K_^-^ m_K_^+^)) unless otherwise stated.

### Trypan blue binding assay

Cells were grown in CTT to a density of 7 × 10^8^ cells/ml, harvested and resuspended in 1% CTT or MC7 buffer to a calculated density of 7 × 10^9^ cells/ml. 20 μl aliquots of the cell suspensions were placed on 0.5% agar supplemented with 0.5% CTT and 20 μg/ml trypan blue or on TPM agar supplemented 20 μg/ml trypan blue. Plates were incubated at 32°C for 24 hrs and then visualized using a Leica MZ8 stereomicroscope and imaged using Leica DFC280 camera.

### c-di-GMP quantification

Quantifications of c-di-GMP levels in starving *M*. *xanthus* cells were performed as described [45]. Briefly, exponentially growing cells were harvested from CTT growth medium and resuspended to a cell density of 10^9^ cells/ml in MC7 (10 mM MOPS pH 7.0, 1 mM CaCl_2_) starvation buffer, and incubated in submerged culture on a solid surface or in suspension with shaking. At the indicated time points, cells were harvested at 4°C, 2500× g, 20 min. Cells were lysed in extraction buffer (HPLC grade acetonitrile/methanol/water (2/2/1, v/v/v)), supernatants pooled and evaporated to dryness in a vacuum centrifuge. Pellets were dissolved in HPLC grade water for analysis by LC-MS/MS. All experiments were done in biological triplicates. For all samples, protein concentrations were determined in parallel using a Bradford assay (Bio-Rad).

### qRT-PCR analysis

Total RNA was isolated from cells developed in submerged culture using a hot-phenol extraction method as described [70]. RNA was treated with DNase I (Ambion) and purified with the RNeasy kit (Qiagen). RNA was confirmed to be free of DNA by PCR analysis. 1 μg of RNA was used to synthesize cDNA with the High capacity cDNA Archive kit (Applied Biosystems) using random hexamers primers. qRT-PCR was performed in 25 μl reaction volume using SYBR green PCR master mix (Applied Biosystems) and 0.1 μM primers specific to the target gene in a 7300 Real Time PCR System (Applied Biosystems). Relative gene expression levels were calculated using the comparative Ct method. All experiments were done with two biological replicates each with three technical replicates.

### Immunoblot analysis

Immunoblots were carried out as described [69]. Rabbit polyclonal α-PilA [50], α-PilC [71] and α-DmxB antibodies were used together with horseradish-conjugated goat anti-rabbit immunoglobulin G (Sigma) as secondary antibody. Blots were developed using Luminata crescendo Western HRP Substrate (Millipore). T4P were sheared from cells developed in submerged culture, purified, followed by immunoblot analyses with α-PilA antibodies as described [50]. To generate rabbit, polyclonal α-DmxB antibodies, purified DmxB-His_6_ was used to immunize rabbits using standard procedures [69].

### Protein purification

For expression and purification of His_6_-tagged proteins, proteins were expressed in *E*. *coli* Rosetta 2(DE3) (F^-^ *ompT hsdS*_B_(r_B_^-^ m_B_^-^) *gal dcm* (DE3) pRARE2) at 18°C or 37°C. His_6_-tagged proteins were purified using Ni-NTA affinity purification. Briefly, cells were resuspended in buffer A (50 mM Tris-HCl, 150 mM NaCl, 10 mM imidazole, 1 mM DTT, 10% glycerol, pH 8) and lysed using a French pressure cell. To purify DmxB variants and PmxA, after centrifugation (1 hr, 48000× g, 4°C) lysates were loaded on a Ni-NTA agarose column (Qiagen) and washed with 20x column volume using buffer B (50 mM Tris-HCl, 300 mM NaCl, 20 mM imidazole, pH 8). Proteins were eluted with buffer C (50 mM Tris-HCl, 300 mM NaCl, 200 mM imidazole, pH 8). To purify EpsI/Nla24, 1 ml HiTrap chelating HP columns (GE Healthcare, Life Sciences) were equilibrated with 10 volumes of washing buffer (20 mM HEPES pH 7.5, 250 mM NaCl, 2 mM MgCl_2_, and 2.5% (v/v) glycerol pH 6.8) and loaded with cell lysate. Following protein immobilization, the column was washed with 10 volumes of washing buffer containing 50mM imidazole, before proteins were eluted using washing buffer containing 500mM imidazole.

### *In vitro* DGC and PDE assays

DGC and PDE activities were determined as described [72, 73]. Briefly, assays were performed with 10 μM of purified proteins (final concentration) in a final volume of 40 μl. Reaction mixtures were pre-incubated for 5 min at 30°C in reaction buffer (50 mM Tris-HCl pH 8.0, 300 mM NaCl, 10 mM MgCl_2_). DGC reactions were initiated by adding 1 mM GTP/[α-^32^P]-GTP (0.1 μCi/μl) and incubated at 30°C for the indicated periods of time. PDE reactions were initiated by adding ^32^P-labeled c-di-GMP. Reactions were stopped by addition of one volume 0.5 M EDTA. Reaction products were analyzed by polyethyleneimine-cellulose TLC chromatography as described [12]. Plates were dried prior to exposing a phosphor-imaging screen (Molecular Dynamics). Data were collected and analyzed using a STORM 840 scanner (Amersham Biosciences) and ImageJ 1.46r, respectively.

### Preparation of [α-^32^P]-labeled c-di-GMP

[α-^32^P]-labeled c-di-GMP was prepared by incubating 10 μM His_6_-DgcA^WT^ (final concentration) with 1 mM GTP/[α-^32^P]-GTP (0.1 μCi/μl) in reaction buffer (50 mM Tris-HCl pH 8.0, 300 mM NaCl, 10 mM MgCl_2_) in a total volume of 200 μl overnight at 30°C. The reaction mixture was then incubated with 5 units of calf intestine alkaline phosphatase (Fermentas) for 1 hr at 22°C to hydrolyze unreacted GTP. The reaction was stopped by incubation for 10 min at 95°C. The reaction was centrifuged (10 min, 15000× g, 20°C) and the supernatant used for the PDE assay.

### *In vitro* c-di-GMP binding assay

In the DRaCALA [48, 74] [α-^32^P]-c-di-GMP was mixed with 20 μM of the relevant protein and incubated for 10 min at 22°C in binding buffer (10 mM Tris, pH 8.0, 100 mM NaCl, 5 mM MgCl_2_). 10 μl of this mixture was transferred to a nitrocellulose filter (GE Healthcare), allowed to dry and imaged using a STORM 840 scanner (Amersham Biosciences). For competition experiments, 0.4 mM unlabelled c-di-GMP (Biolog) or GTP (Sigma) was used. In the SPR-based method [55], experiments were done at 25°C with a Biacore T200 system (GE Healthcare) using a Streptavidin SA sensor chip (GE Healthcare), which has four flow cells each containing Streptavidin pre-immobilized to a carboxymethylated dextran matrix. Flow cell (FC) one (FC1) and FC3 were kept blank to use for reference subtraction. To remove unconjugated Streptavidin, the chip was washed three times with 1 M NaCl, 50 mM NaOH. 100 nM biotinylated c-di-GMP (BioLog) was immobilised on FC2 and FC4 of the Streptavidin SA chip at a 50 RU immobilisation level with a flow rate of 5 μl/min. Purified, soluble EpsI/Nla24-His_6_ was prepared in SPR buffer (10 mM HEPES, 150 mM NaCl, 0.1% (v/v) Tween 20, 2 mM MgCl_2_, pH 6.8). Samples were injected with a flow rate of 5 μl/min over the four flow cells for 90 sec followed by buffer flow for 60 sec. The chip was washed at the end of each cycle with 1 M NaCl. An increasing range of protein concentrations (62.5 nM, 125 nM, 250 nM, 500 nM, 1.0 μM, 2.0 μM, 4.0 μM) was used, with replicates for certain protein concentrations as appropriate. Sensorgrams were analysed using Biacore T200 BiaEvaluation version 1.0 (GE Healthcare). Data were plotted using Microsoft Excel and GraphPad Prism. The experiment was repeated three times independently.

### Biotinylated c-di-GMP pull-down

*E*. *coli* whole cell lysates before and after induction (0.5mM IPTG for 5 hrs at 28°C) of EpsI/ Nla24 were prepared by sonication. The lysed cells were centrifuged (1 hr, 13000× g, 4°C) and 45 μl of the soluble fraction was collected and mixed with biotinylated c-di-GMP (BioLog B098) at a final concentration of 30 μM. The mixture was incubated O/N on a rotary wheel at 8°C. The next day, UV cross-linking was carried out using a UV Stratalinker (Stratagene) for 4 min on ice, to stabilise c-di-GMP/protein complexes. 25μl of Streptavidin magnetic beads (Invitrogen) were added to the mixture, and incubated for 1 hr on a rotary wheel at 8°C. A magnet was used to isolate the Streptavidin magnetic beads and five washing steps were carried out using 200 μl of the protein wash buffer each time (20 mM HEPES pH 7.5, 250 mM NaCl, 2 mM MgCl_2_, and 2.5% (v/v) glycerol pH 6.8), to remove non-bound proteins. The washed Streptavidin beads were resuspended in 15 μl wash buffer, 4× SDS loading buffer was added, incubated at 95°C for 10 min and then loaded on a 12% SDS-PAGE protein gel. The gel was then developed using InstantBlue (Expedeon).

## Supporting Information

S1 Fig Systematic analysis of *M*. *xanthus* genes encoding proteins containing a GGDEF, EAL or HD-GYP domain. Cells were treated and spores enumerated as described in Fig 2B. Scale bars: TPM agar 500 μm, submerged culture 100 μm. Strains indicated in red have defects in development only.
(EPS)

S2 Fig Bioinformatics analysis of actA gene and ActA protein. (A) Flanking genome region of *actA*. Old and new annotation of *actA* gene is indicated in black. Red line marks the region deleted from *actA* in strain DK10605 [46]. Green line marks the region deleted from *actA* in strain SA3599 used in this work. Numbers indicate position in bp relatively to the first nucleotide in the newly annotated *actA* (+1). Direction of gene transcription is indicated by the arrows. (B) Sequence comparison of ActA homologs. The first two lines indicate the old and new annotation of ActA, respectively. From top to bottom: ActA from *M*. *xanthus*, *M*. *fulvus*, *M*. *stipitatus*, *Corallococcus coralloides* and *Stigmatella aurantiaca*.
(EPS)

S3 Fig Genetic neighborhood of *MXAN3735* (*dmxB*) and phenotype of Δ*MXAN3734* mutant during development. (A) *dmxB* locus. Direction of transcription of *dmxB* (*MXAN3735*) and genes flanking *dmxB* are indicated by the arrows. Numbers indicate position in bp relatively to start of *dmxB* (+1). Domain structure of proteins encoded by *dmxB* and flanking genes is indicated on the right. Domain structure was analyzed using [47]. (B) Cells of the indicated genotypes were treated and spores enumerated as described in Fig 2B. Scale bars, TPM agar 500 μm, submerged culture 100 μm.
(EPS)

S4 Fig qRT-PCR analysis of *eps* gene transcription in WT and the Δ*dmxB* mutant during development. (A) The *eps* locus in *M*. *xanthus*. Analyzed genes are indicated (red arrows) in the schematic of the *eps* locus together with the predicted function of the corresponding proteins indicated. The five genes in the dashed box have been suggested to form an operon [41]. (B) Total RNA was isolated at the indicated time points from cells of WT (closed circles) and the Δ*dmxB* mutant (open squares) developed in submerged culture. Transcript levels are shown as mean ± standard deviation from two biological replicates with each three technical replicates relative to WT at 0 hrs.
(EPS)

S5 Fig Domain structure of EpsI/Nla24 and sequence comparison of AAA+ domains of EpsI/Nla24, FleQ and VpsR. Domain structure was analyzed using [47]. Conserved features important for AAA+ activity (Walker A, Walker B, R finger) and interaction with σ^54^ (Loop L1, Loop L2) are indicated in different colors. c-di-GMP binding motifs identified in FleQ [59] are marked by red boxes, conserved residues in these motifs as identified in [59] are in bold. Red arrows below the alignment indicate conserved Arg residues in FleQ important for c-di-GMP binding, black arrows indicate other c-di-GMP-interacting residues identified in the FleQ structure [59].
(EPS)
